# A Novel Alkaliphilic *Streptomyces* Inhibits ESKAPE Pathogens

**DOI:** 10.3389/fmicb.2018.02458

**Published:** 2018-10-16

**Authors:** Luciana Terra, Paul J. Dyson, Matthew D. Hitchings, Liam Thomas, Alyaa Abdelhameed, Ibrahim M. Banat, Salvatore A. Gazze, Dušica Vujaklija, Paul D. Facey, Lewis W. Francis, Gerry A. Quinn

**Affiliations:** ^1^Institute of Life Sciences, Swansea University Medical School, Swansea, United Kingdom; ^2^School of Biomedical Sciences, Ulster University, Coleraine, United Kingdom; ^3^Laboratory for Molecular Genetics, Ruđer Bošković Institute, Zagreb, Croatia

**Keywords:** alkaliphile, antimicrobial, *Streptomyces*, ESKAPE pathogens, multi-resistant, ethnopharmacology

## Abstract

In an effort to stem the rising tide of multi-resistant bacteria, researchers have turned to niche environments in the hope of discovering new varieties of antibiotics. We investigated an ethnopharmacological (cure) from an alkaline/radon soil in the area of Boho, in the Fermanagh Scarplands (N. Ireland) for the presence of *Streptomyces*, a well-known producer of antibiotics. From this soil we isolated a novel (closest relative 57% of genome relatedness) *Streptomyces* sp. capable of growth at high alkaline pH (10.5) and tolerant of gamma radiation to 4 kGy. Genomic sequencing identified many alkaline tolerance (antiporter/multi-resistance) genes compared to *S. coelicolor* M145 (at 3:1), hence we designated the strain *Streptomyces* sp. myrophorea, isolate McG1, from the Greek, myro (fragrance) and phorea (porter/carrier). *In vitro* tests demonstrated the ability of the *Streptomyces* sp. myrophorea, isolate McG1 to inhibit the growth of many strains of ESKAPE pathogens; most notably carbapenem-resistant *Acinetobacter baumannii* (a critical pathogen on the WHO priority list of antibiotic-resistant bacteria), vancomycin-resistant *Enterococcus faecium*, and methicillin-resistant *Staphylococcus aureus* (both listed as high priority pathogens). Further *in silico* prediction of antimicrobial potential of *Streptomyces* sp. myrophorea, isolate McG1 by anti-SMASH and RAST software identified many secondary metabolite and toxicity resistance gene clusters (45 and 27, respectively) as well as many antibiotic resistance genes potentially related to antibiotic production. Follow-up *in vitro* tests show that the *Streptomyces* sp. myrophorea, isolate McG1 was resistant to 28 out of 36 clinical antibiotics. Although not a comprehensive analysis, we think that some of the Boho soils’ reputed curative properties may be linked to the ability of *Streptomyces* sp. myrophorea, isolate McG1 to inhibit ESKAPE pathogens. More importantly, further analysis may elucidate other key components that could alleviate the tide of multi-resistant nosocomial infections.

## Introduction

The global increase in multi-resistant ESKAPE pathogens (*Enterococcus faecium*, *Staphylococcus aureus*, *Klebsiella pneumoniae*, *Acinetobacter baumannii*, *Pseudomonas aeruginosa*, and *Enterobacter* species) has created an urgent need to develop replacement therapies. ESKAPE pathogens are responsible for the top 6 health care-associated infections (HAIs) and many have been identified as priority antibiotic-resistant bacteria (Jelic et al., 2016; Santajit and Indrawattana, 2016; Founou et al., 2017; Tacconelli et al., 2018). Infections with multi-resistant pathogens are extremely hard to treat and may spread throughout a hospital or community environment (Jelic et al., 2016; Santajit and Indrawattana, 2016). It was thought that developing new antibiotics from combinatorial chemistry would be able to eliminate these resistant bacteria. However, years of trials have not produced anything like the number of new drugs necessary to stem the tide of multi-resistant bacteria (Lewis, 2013). In addition, the production of new antibiotics tapered off in the early 1980s due to unfavorable market conditions which has led to a crisis in the supply line of new antibiotics (Santajit and Indrawattana, 2016; Founou et al., 2017). Normally, bacterial infections are treated with the simplest, most effective antibiotics, however, multi-resistant pathogens usually require treatment with higher tier antibiotics or antibiotics of last resort (Santajit and Indrawattana, 2016). There are no guarantees of success with these treatments and they can involve expensive and sometimes toxic chemotherapy. If all solutions fail, infections by ESKAPE pathogens can lead to death of the patient and a spread of multi-resistant strains. Antibacterial resistance has now been detected for nearly all new antibiotics, even those of last resort (Jelic et al., 2017; Li and Webster, 2018). As a consequence, the WHO have created an urgent priority list for discovery of new antibiotics (Tacconelli et al., 2018).

Current strategies to alleviate the shortage of new antibiotics have turned to niche environments such as deserts, thermal vents, and alkaline environments in the hope that they might produce exotic varieties of current antibiotics (Sato et al., 1983; Mao et al., 2007; Yucel and Yamac, 2010; Mohammadipanah and Wink, 2015). Alkaline environments in particular have proven to be a rich source of antibiotics, many derived from *Streptomyces* bacteria (Sato et al., 1983; Yucel and Yamac, 2010; Behroozian et al., 2016; Maciejewska et al., 2016). Together with other members of the phylum Actinobacteria, *Actinomycetes*, are responsible for the synthesis of more than half of modern medicines including antimicrobial, anti-cancerous (Noomnual et al., 2016), antiviral (Yokomizo et al., 1998), antifungal (Nguyen and Kim, 2015), and antiparasitic compounds (Procopio et al., 2012). In 1943, it was streptomycin (from *Streptomyces*) that was the great savior against the formally incurable scourge of tuberculosis (Schatz et al., 1944).

Another promising avenue of drug (re)discovery lies in the investigation of traditional medicines or ethnopharmacology. Although ancient medical traditions are well known in Chinese and Native American cultures, less is known about European folk medicines (Foley, 2015; Behroozian et al., 2016; Kung et al., 2018). One of the last vestiges of continuous ethnopharmacological culture can be found on the most westerly fringes of Europe, in rural locations on the island of Ireland (Foley, 2015). One such cure originates from a region of the West Fermanagh Scarplands known locally as Boho (pronounced Bo) in Northern Ireland. This cure is derived from an alkaline soil deposited in the late Pleistocene period (circa 9,126,000–11,700 years ago) on a bedrock of Carboniferous Dartry limestone (circa 335 million years ago) imparting an alkaline/high radon character to the soil (Brunton and Mason, 1979). Traditionally, this cure had been used to treat a variety of conditions from toothache to infections by placing a small portion of the soil wrapped in cloth next to the infection or underneath the users’ pillow for 9 days. The soil was then returned to the area of sampling. The exact specificity and origins of the cure are obscured by lack of documentation, however, some relatively recent written records remain, associating it with the grave of James McGirr, a cleric and healer who died in 1815 (Gallachair, 1975). Previous to this time the area had significance as an amphitheater for the Druids and a symbolic place for Neolithic peoples as evidenced by the nearby Reyfad stones (Halpin and Newman, 2009).

The purpose of this paper is to report the isolation of a novel alkaliphilic strain of *Streptomyces* from soil with antimicrobial activity against multiresistant ESKAPE pathogens which may have potential clinical applications.

## Materials and Methods

### Sampling

The Boho soil sample was collected from an alkaline escarpment region (Latitude-54.364637°N Longitude-7.820939°W) at the Sacred Heart Church, in the townland of Toneel North, Boho, Fermanagh, United Kingdom (Donnelly et al., 2003) on the 28 July 2015. The test soil, which was pre-aliquoted into small cloth bags on site, was sampled with a sterile spatula. Instructions as to the traditional uses and practices with the soil are displayed on the door of an adjacent building. Approximately 25 g of this soil sample was collected in a sterile conical sample tube (50 ml) and dispatched to the laboratory for analysis. For laboratory analysis, 1 g of the Boho soil sample was diluted in 1 ml sterile water, vortexed, and cultured on International Streptomyces Project (ISP) 2 agar (1/5th strength), and Starch agar (1/5th strength) for the initial *Streptomyces* isolation.

### Microorganism Strains

*Escherichia coli* (ATCC, K12-MG1655) was provided by Dr. D. Zahradka, Ruđer Bošković Institute (RBI), Zagreb, Croatia.

*Bacillus subtilis* (strain 168) was provided by Dr. D. Vujaklija (RBI, Croatia).

ESKAPE pathogens listed below and other ATCC strains listed were provided by Dr. M. Jelić (Jelic et al., 2016, 2017).

*Enterococcus faecium* – strains: a, b, c (VRE), d, e, and f.

*Staphylococcus aureus* – strains: a, b (MRSA), c, d, and e (MRSA).

*Klebsiella pneumoniae* – strains: a, b, c, and d.

*Acinetobacter baumannii* – strains: a, b, c, d, and e.

*Pseudomonas aeruginosa* – strains: a, b, c, and ATCC 27853.

*Enterobacter cloacae* – strain: a.

The ESKAPE pathogens were clinical isolates collected through regular hospital activities. Species identification was performed using standard biochemical methods (tests) and the VITEK 2 system (bioMérieux, France) (Jelic et al., 2016, 2017). Isolates were assigned unique isolate IDs and subsequently anonymized with designations a, b, c, etc. (meaning the strains cannot be linked to patients in any identifiable manner) in accordance with European regulations. Stocks of original *Streptomyces* were frozen at -80°C in 18% glycerol after their initial isolation.

### Microbiological Media

Microbiological media used in these experiments: ISP 2 media (1/5th strength: meaning 20% of standard ingredients except agar which was 2%) and Starch media (1/5th strength) for initial *Streptomyces* isolation. Soy Flour Mannitol (SFM) for *Streptomyces* sub culture and growth. Alkaline SFM for selection of alkaline tolerant *Streptomyces* (soy flour 10 g, mannitol 10 g, agar 20 g, CaCO_3_ 1 g, humic acid 0.002 g, pH adjusted to 8.3 before sterilization). Blood agar was used for the isolation and cultivation of clinical isolates and Mueller-Hinton agar used for antimicrobial tests unless organisms specifically required enriched blood media.

For the determination of *Streptomyces* alkaline tolerance, we used ISP-2 media supplemented with *Streptomyces* minor elements solution [consisting of 0.1% (wt/vol) (each) of ZnSO_4_⋅7H_2_O, FeSO_4_⋅7H_2_O, MnCl_2_⋅4H_2_O, and CaCl_2_ anhydrous], 1% (wt/vol) glucose, and 0.02% (vol/vol) NaH_2_PO_4_–K_2_HPO_4_ buffer (0.1 M, pH 6.8). It was necessary to increase the concentration of agar to 4% after pH 13.2, and 6% after 13.4, due to the inability of lower agar concentrations to solidify. The pH of the agar was adjusted after sterilization (when the agar had cooled but was still liquid, i.e., 42–45°C) by the addition of appropriate volumes of filter sterilized sodium carbonate buffer (0.2 M Na_2_CO_3_ + 0.2 M NaH_2_CO_3_; pH 9.2 → 10.7) and potassium chloride/sodium hydroxide buffer (0.2 M KCl + 0.2 M NaOH; pH 11.6 → 13.0). The pH of the agar was checked immediately after the agar had set.

On the occasions when the robust growth of frozen stocks of *Streptomyces* isolates seemed to decline (as was observed from 2-year-old stocks, even when stored in glycerol at -80°C), stocks were revived by cultivation of the *Streptomyces* sp. myrophorea, isolate McG1 on ISP 2 agar (1/5th) supplemented with *Streptomyces* minor elements solution. In some cases, a mineral solution was made from alkaline soil by dissolving 1 g soil in 1 g water, vortexed (1-min), centrifuged (15,616 ×*g*, 10 min) to clear the supernatant and then added at a concentration of 0.5% (vol/vol) to pre-solidified agar. In both cases supplements were filter sterilized (syringe filter, 0.2 μm) prior to their addition to the agar to prevent precipitation of ferric compounds. Cores of this agar were used as negative controls in antimicrobial inhibition assays involving Mueller-Hinton agar. All reagents were supplied by Oxoid (Basingstoke, Hampshire, United Kingdom) except agar which was supplied by Melford (Melford, Suffolk, United Kingdom).

### Atomic Force Microscopy

Atomic Force Microscopy (Bruker BioScope Catalyst; Bruker Instruments, Santa Barbara, CA, United States) was used to visualize *Streptomyces* hyphae growing on glass cover slips. The measurements were conducted in air, using TESPA cantilevers (Bruker Instruments, Santa Barbara, CA, United States) in Tapping Mode, with a nominal spring constant of 40 N/m and a nominal resonant frequency of 300 kHz. Off-line processing involved first-order plane fitting and flattening using the software Nan scope Analysis 1.50 (Bruker Instruments, Santa Barbara, CA, United States).

### pH Measurement

Soil pH was measured by dissolving 1 g of soil in 5 ml distilled water, shaking for 5 min and then waiting 1 h for the soil to settle (measuring in triplicate using a pH-meter; Mettler-Toledo Seven Compact, Leicester, United Kingdom). A similar procedure was used with pH paper, where 1 g of soil was dissolved in 2 ml distilled water, vortexed for 15 min, and applied in small aliquots to pH indicator strips (EMD Millipore ColorpHast, Burlington, MA, United States). The pH strips were accurate to 0.5 pH units.

To test the alkaline tolerance of bacteria, a pH gradient was established from pH 9.0 to 13.0 in ISP-2 by adding sterilized buffer as described above. The pH of agar surface was continuously monitored using flat pH sticks (Macherey-Nagel GmbH & Co. KG, Düren, Germany) as described (Jones et al., 2017). The pH strip accuracy was routinely tested against standard pH calibration solutions.

### Gamma-Irradiation of *Streptomyces* sp. Myrophorea, Isolate McG1 Spores

*Streptomyces* sp. myrophorea, isolate McG1 spore concentrations were estimated by cultivation of dilutions of the spore stock. A 1 ml spore suspension (estimated to be 1 × 10^5^ spores/ml) received gamma radiation doses of 0.25, 0.5, 1.0, 2.0, 4.0, 10.0, 15.0, and 20.0 kiloGray (kGy) from a cobalt source (^60^Co, 8.2645 Gy/s). Spore suspensions were contained in 2 ml plastic microfuge tubes surrounded by a layer of ice inside a polystyrene ice container. After irradiation, aliquots of the spore suspensions were spread (100 μl) on SFM agar and cultivated for 2–3 weeks. The growth of one colony (colony-forming units = CFU) was interpreted to be the survival of one spore.

### Antimicrobial Tests

#### Agar Overlay

A standard agar overlay combined with an antibiotic assay was used to test the inhibitory potential of *Streptomyces* sp. myrophorea, isolate McG1 against ESKAPE pathogens (Nkanga and Hagedorn, 1978; Lehrer et al., 1991). Briefly, wells were made in a base layer (15 ml) of Mueller-Hinton agar. Control wells contained standard amounts of dissolved antibiotics were allowed to absorb into the agar over a period of 2 h. Once the wells were dry, an agar core of *Streptomyces* sp. myrophorea, isolate McG1 (cultivated for 9 days 20°C) was placed in an empty well in addition to a negative control of the original media without *Streptomyces* sp. myrophorea, isolate McG1. This base layer was then overlaid with 15 ml Mueller-Hinton agar (cooled to 43°C) that incorporating the test organisms (ESKAPE pathogens with a minimum 5 × 10^5^ CFU/ml). Bacterial inhibition was indicated by a clear zone in the confluent growth of the test organism after overnight incubation.

### Kirby–Bauer

Antibiotic sensitivity tests (antibiograms) for the *Streptomyces* sp. myrophorea, isolate McG1 and for ESKAPE pathogens followed the Kirby–Bauer protocol (Bauer et al., 1966). The resistance profiles (antibiograms) of clinical isolates (ESKAPE pathogens) were determined using standard concentrations of antibiotic impregnated discs (Oxoid) as proposed by the European Committee on Antimicrobial Susceptibility Tests (EUCAST) (**Supplementary Table S1**). Breakpoint tables for interpretation of MICs and zone diameters can be found in Version 8.0, 2018^1^.

### Genotypic Characterization of *Streptomyces* sp. Myrophorea, Isolate McG1

#### Genome Sequencing

Cultures of *Streptomyces* sp. myrophorea, isolate McG1 were grown on 1/10th strength LB agar for DNA extraction using a Qiagen DNA mini kit (Qiagen, MD, United States) with the inclusion of a lysis step using lysozyme. Genomic DNA was prepared for sequencing using Qiagen FX and sequenced on an Illumina MiSeq platform using a 600 cycle V3 reagent kit.

#### Assembly and Annotation of Genome

Paired-end reads were subjected to quality filtering using the Trimmomatic tool (4 bp sliding window of Q20) (Bolger et al., 2014) prior to *de novo* genome assembly using SPAdes under default parameters (Bankevich et al., 2012). The genome assembly was assessed using QUAST (Gurevich et al., 2013) and annotated using Prokka (Seemann, 2014) and Rapid Annotation using Subsystem Technology (RAST)^2^ (Aziz et al., 2008).

### Phylogenetic Analysis

*In silico* DNA–DNA hybridization was performed using the Genome–Genome Distance Calculator (GGDC) v 2.1 (Auch et al., 2010) using all *Streptomyces* genomes (868 sequences) as references. All genomes were downloaded from GenBank^3^.

Phylogenetic placement of *Streptomyces* sp. myrophorea, isolate McG1 was performed using PhyloPhlAn (Segata et al., 2013). Protein sequences from annotated *Streptomyces* genomes were retrieved autonomously from the GenBank FTP site using the term “*Streptomyces*” as a query. Ortholog identification and alignment was performed in Phylophlan. A maximum-likelihood phylogeny was reconstructed from the concatenated alignments in FastTree MP (JTT + CAT) implemented in the Cipres Science Gateway Server (Miller et al., 2010). The robustness of the phylogeny was assessed using 1000 bootstrap pseudoreplicates.

### Secondary Metabolite Analysis

Gene clusters known to be involved in secondary metabolite biosynthesis, self-immunity, or resistance were identified using Antibiotics and Secondary Metabolite Analysis Shell (anti-SMASH) version 4.0.0 (Medema et al., 2011). The GenBank sequence file (from Prokka annotation) was submitted to the web interface selecting all extra features of annotation.

## Results

### Isolation of *Streptomyces* From Soil

**FIGURE 1:**
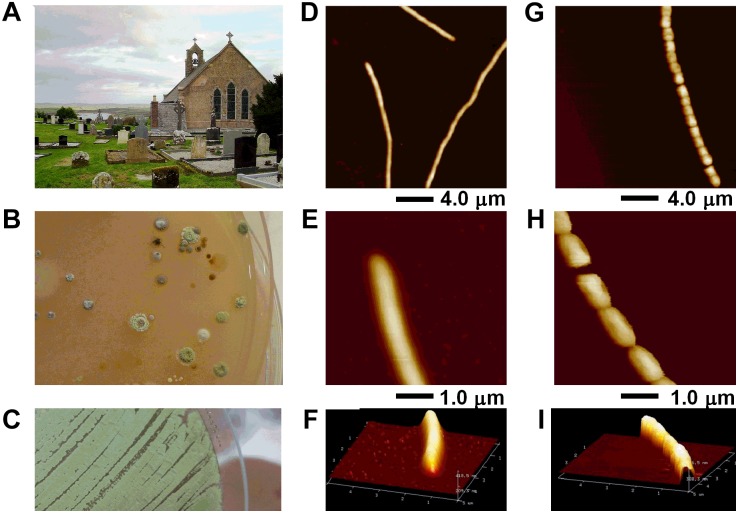
*Streptomyces* sp. myrophorea isolation from **(A)** Boho soil sample site, **(B)** selective enrichment agar, and **(C)** pure culture. **(D–I)** AFM data of *Streptomyces* sp. myrophorea, isolate McG1 grown on glass coverslips and imaged in air by Tapping Mode. Both vegetative **(D–F)** and sporulating **(G–I)** stages are shown. Hyphae overviews are shown in **D** and **G**, while higher resolution scans in **E** and **F** for vegetative hyphae, and in **H** and **I** for sporulating hyphae.

Aliquots (20 μl) of diluted soil samples were cultivated on several agars to select for *Streptomyces* including ISP2 (1/5th) and alkaline SFM (**Figure 1**). The original soil was returned to the sampling site as per local tradition.

Preliminary screening of the Boho soil sample resulted in the isolation of eight (visually different) *Streptomyces*-like colony types as determined by colony morphology and growth characteristics. The *Streptomyces* isolate which had the most consistent inhibitory activity toward Gram-positive and Gram-negative bacteria (initially labeled as *Streptomyces* sp. myrophorea, isolate McG1) was selected for further characterization and testing (**Figure 1**).

### *Streptomyces* Characterization

Visually *Streptomyces* sp. myrophorea, isolate McG1 had (powdery) light to dark green colonies on SFM agar with green to very light green/white spores (**Figures 1B,C**). After a period of approximately 3–5 days, colonies emitted a distinctly “germaline” odor. *Streptomyces* sp. myrophorea, isolate McG1 appeared to be a non-motile, spore forming bacteria with very slender vegetative and aerial hyphae. Atomic force microscopy revealed that the bacterial hyphae were approximately 0.5–1.0 μm width, with spores in a linear conformation (17–20 spores) having a width of approximately 0.5–1.0 μm (**Figures 1D–I**).

### Alkaline Tolerance of *Streptomyces* sp. Myrophorea, Isolate McG1

The Boho soil sample had an average pH of 7.8 (mean ± standard deviation 0.35, by pH meter). Parallel measurements using pH strips indicated a wider range of pH from pH 7.8 to pH 8.5 (six readings at two different time points).

**FIGURE 2:**
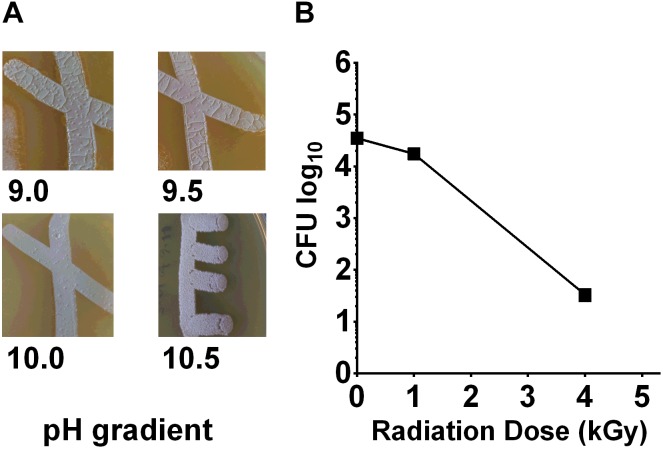
*Streptomyces* sp. myrophorea, isolate McG1 development on pH and irradiation gradients. **(A)** *Streptomyces* sp. myrophorea, isolate McG1 cultivated on ISP-2 agar under increasing pH. Images indicate final measurements, pH 9.0 (originally pH 9.5), pH 9.5 (originally pH 10.0), pH 10.0 (originally pH 10.7), and pH 10.5 (originally pH 11.6). Images are representative of three independent experiments. **(B)** Effect of *γ-*irradiation (^60^Co) on spore survival of *Streptomyces* sp. myrophorea, isolate McG1. All tests performed in triplicate. The variation in spore survival is indicated using standard error of the mean.

To measure the pH tolerance of *Streptomyces* sp. myrophorea, isolate McG1, a pH gradient was prepared as described. The pH levels of the agar surface were measured after it had set and every subsequent day after this using flat pH sticks (see the section “Materials and Methods”). The results revealed pronounced *Streptomyces* sp. myrophorea, isolate McG1 growth around pH 9.0 to pH 10 after 3–4 days. Growth was slower when bacteria were inoculated on agar that was prepared at pH 11.5 and took 5 days to be visible, however, during this time the pH of the agar also dropped to pH 10.5 (**Figure 2A**). In addition, we observed that *Streptomyces* sp. myrophorea, isolate McG1 inoculated at pH 12.2 or 13.0 could survive several days without prominent growth. As above, we noted that bacterial biomass became visible once the pH of the agar decreased to pH 10.5. In contrast *S. coelicolor* M145 was unable to grow in high alkaline conditions (pH 9 and above) implying that our new strain of *Streptomyces* was more alkaliphilic.

In contrast to the alkaline conditions, *Streptomyces* sp. myrophorea, isolate McG1 did not grow well under acidic conditions, the lowest pH for growth being pH 6.5.

Based on changes in pH during sterilization of media and between buffered and unbuffered media, we realize that it is very important to measure the pH of the agar surface throughout the bacterial growth cycle.

### Radio-Tolerance of *Streptomyces* sp. Myrophorea, Isolate McG1

The alkaline environment of the Boho area is not the only characteristic which makes this region a niche habitat. The (limestone/shale) bedrock also releases radon gas which can be found at levels as high as 710 bq/m^3^ (domestic dwelling annual totals) in Boho and adjacent areas (Daraktchieva et al., 2015).

To test the effects of radiation on the survival of *Streptomyces* sp. myrophorea, isolate McG1, we subjected spore solutions to increasing levels of gamma radiation, i.e., spores were removed from a chamber after an exposure to absorbed doses of 0.25 → 20 kGy. After irradiation, aliquots (100 μl) of spores were spread evenly on diluted ISP2 agar in triplicate. The agar plates were then incubated at 4–10°C for 3 weeks before enumeration. Our measurements indicated that spores of *Streptomyces* sp. myrophorea, isolate McG1 were able to tolerate doses of 4 kGy of gamma irradiation and still remain viable (**Figure 2B**). In comparison, another *Streptomyces*, *Streptomyces radiopugnans* resists radiation expose of up to 15 kGy, whereas vegetative cells such as *Deinococcus radiodurans* tolerates an exposure of 12 kGy, *E. coli* 600 Gy, and human cells 4 Gy (Mao et al., 2007; Daly, 2012).

### Antimicrobial Tests

To test antimicrobial potential of *Streptomyces* sp. myrophorea, isolate McG1, agar cores from the *Streptomyces* were embedded in Mueller-Hinton test agar and overlaid with a suspension of ESKAPE pathogens. The pathogens were isolated from different hospitals in Croatia based on clinical antimicrobial susceptibility data. Controls consisted of ampicillin (Amp-20 μg), chloramphenicol (Cam-20 μg), ciprofloxacin (Cip-5 μg), gentamicin (Gen-30 μg), kanamycin (Kan-10 μg), streptogramin (10 μg), and ampicillin + sulbactam (Amp + Sulf-10 μg + 10 μg). An agar core from the original ISP2 supplemented agar was used as a negative control.

ESKAPE pathogens were considered susceptible (S) to antibiotics if the zone of inhibition was greater than 12 mm diameter; resistant (R), if zone was less than 8 mm radius and of intermediate status (I) if the zone was between 8 and 12 mm or there were a few colonies appearing in between the beginning of the zone and the edge of the antibiotic disc. A single mark indicates uniformity of result in a triplicate. Tests with variable results are indicated by the result of each replicate, i.e., S/R/S indicates the result of 1st/2nd/3rd test (**Table 1**).

**Table 1:** Inhibitory effects of *Streptomyces* sp. myrophorea, isolate McG1 against ESKAPE pathogens.

Bacteria	*Streptomyces* sp. myrophorea, isolate McG1	Antibiotic sensitivity	
*E. faecium*			
Strain (a)	S	Amp R,	Cam S
Strain (b)	S	Amp S,	Cam R
Strain (c) (VRE)^∗∗^	S	Amp R,	Cam R
Strain (d)±	R	Amp S,	Cam S
Strain (e)±	R	Amp S,	Cam S
Strain (f)±	R	Amp R,	Cam S
*S. aureus*			
Strain (a)	S	Amp S/S/R,	Cam S
Strain (b) MRSA^∗∗^	S	Amp R,	Cam S
Strain (c)	S	Amp S,	Cam S
Strain (d)	S	Kan S,	Cam S/S/R
Strain (e) MRSA ^∗∗^	S	Amp R,	Cam S
*K. pneumoniae*			
Strain (a)	S	Amp S,	Cam S
Strain (b)	S	Amp S,	Cam S
Strain (c)	S	Amp R,	Cam R
Strain (d)	S	Amp R,	Cam S/R/S
*A. baumannii*			
Strain (a)^∗^	S		Cam R
Strain (b)	R	Amp + Sul S,	Cam R
Strain (c)	I	Amp + Sul R,	Cam R
Strain (d)^∗^	S		Cam R
Strain (e)^∗^	S	Amp R,	Cam R
*P. aeruginosa*			
Strain (a)	I/S/I	Streptogramin R,	Cip S
Strain (b)	S	Kan S,	Cam R
Strain (c)	R/S/R	Streptogramin R,	Cip S
Strain 27853 ATCC	R	Gen S,	Cip S
*E. cloacae*			
Strain (a)	S	Amp R,	Cam R
*E. coli* K12	S	Amp S,	Cam S
*B. subtilis* 168	S	Amp S,	Kan S


*The inhibitory effects of Streptomyces sp. myrophorea, isolate McG1 were compared to those of ampicillin (Amp-10 μg), chloramphenicol (Cam-20 μg), ciprofloxacin (Cip-5 μg), gentamicin (Gen-30 μg), kanamycin (Kan-10 μg), streptogramin: ampicillin + sulbactam (Amp + Sulf-10 μg + 10 μg)) and a negative control (an agar core from the original ISP2 supplemented agar) using the agar overlay method. ESKAPE pathogens were considered susceptible (S) to antibiotics if the zone of inhibition was greater than 12 mm diameter; resistant (R), if zone was less than 8 mm radius and of intermediate status (I) if the zone was between 8 and 12 mm or there were a few colonies appearing in between the beginning of the zone and the edge of the antibiotic disc. A single mark indicates uniformity of result in a triplicate. Tests with variable results are indicated by a sign for each replicate, i.e., S/R/S indicates the result of 1st/2nd/3rd test. Could only be cultivated on blood agar (±), WHO critical-priority bacteria (^∗^), and WHO high priority bacteria (^∗∗^).*

**FIGURE 3:**
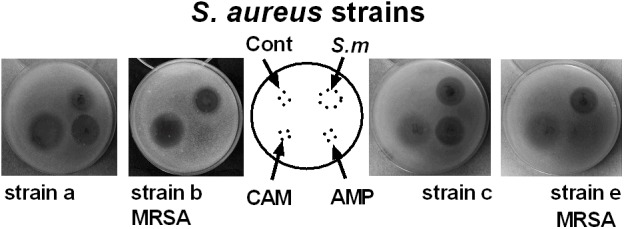
Inhibition of clinical strains of *S. aureus* by agar cores of *Streptomyces* sp. myrophorea, isolate McG1. *S. aureus* (SA) strains: SA strain **a**, SA strain **b** (MRSA, ciprofloxacin resistant), SA strain **c** and SA strain **e** (MRSA, ciprofloxacin sensitive) were cultivated on Mueller-Hinton agar together with agar cores of *Streptomyces* sp. myrophorea, isolate McG1 (S.m). Other test wells contained chloramphenicol (CAM-20 μg), ampicillin (AMP-20 μg), and a negative control of the original ISP-2 agar with mineral supplement (cont). Images are representative of three independent experiments.

ESKAPE pathogens are known to resist many clinical antibiotics including aminoglycosides, beta-lactams, carbapenems and glycopeptides (Founou et al., 2017). Our data on the incubation of ESKAPE pathogens reveal that *Streptomyces* sp. myrophorea, isolate McG1 was broadly inhibitory to both Gram-positive and Gram-negative bacteria. Specifically *Streptomyces* sp. myrophorea, isolate McG1 inhibited carbapenem-resistant *A. baumannii* [listed as a critical pathogen in the WHO priority pathogens list), vancomycin-resistant *E. faecium*, and methicillin-resistant *S. aureus* (which are both listed as high priority on the WHO pathogen list)] and *K. pneumoniae* (**Figure 3** and **Table 1**). Some strains of *E. faecium* that could not be cultivated on Mueller-Hinton agar (they only grew on blood agar) as well as some strains of multi-resistant *P. aeruginosa* were resistant to *Streptomyces* sp. myrophorea, isolate McG1 (**Table 1**).

### Genome Sequencing of *Streptomyces* sp. Myrophorea, Isolate McG1

Whole-genome sequencing of *Streptomyces* sp. myrophorea, isolate McG1 was performed using the Illumina MiSeq system. The total assembled size of the *Streptomyces* sp. myrophorea, isolate McG1 genome was almost 9 MB pairs with a GC content of 71.6%. Parameters predicted by Prokka and QUAST (Gurevich et al., 2013; **Table 2**).

**Table 2:** *Streptomyces* sp. myrophorea, isolate McG1 genome characterization and assembly (Prokka).

Genome characterization	Filtered
Total length (bp)	8,950,068
Number of contigs	110
GC content (%)	71.64
Predicted no. of genes	7643
Number of tRNAs	78
Number of rRNAs	8
N50 score	409,278
Number of Ns per 100 kbp	0


*The “Number of Ns per 100 kbp” is the average number of uncalled bases (Ns) per 100,000 assembly bases, the N50 is defined as the minimum contig length needed to cover 50% of the genome.*

### Deposition of Genome Sequence

The genome sequence was deposited in NCBI under the name of “*Streptomyces* sp. myrophorea, isolate McG1” (TaxID 2099643) or “*Streptomyces* sp. McG1,” Biosample accession number SAMN08518548, BioProject accession number PRJNA433829, Submission ID: SUB3653175, and Locus tag prefix: C4625. The project information is accessible on publication from http://www.ncbi.nlm.nih.gov/bioproject/433829.

*Streptomyces* sp. myrophorea, isolate McG1 was deposited with the National Collection of Type Cultures (NCTC 14177), United Kingdom and the Deutsche Sammlung von Mikroorganismen und Zellkulturen (DSMZ) GmbH, Germany.

### DNA–DNA Hybridization

The top 12 matches against *Streptomyces* genomes and the intergenomic distances for *in silico* DNA–DNA hybridization (DDH) were calculated using GGDC (**Table 3**). The calculation of GGDC Formula 2 is independent of genome length (calculated by dividing found identities by high-scoring pairs and not by whole-sequence length). An absence of any DDH values over the recognized threshold of 70% revealed that our isolate was an uncharacterized species of *Streptomyces*.

**Table 3:** The closest 12 matches to *Streptomyces* sp. myrophorea, isolate McG1 in NCBI (by GGDC) (Formula 2 statistics).

Isolate	Accession number	DDH	Prob. DDH ≥ 70%	Distance
*Streptomyces anulatus* strain ATCC 11523^T^	PRJNA257397	55.1	35.24	0.061
*Streptomyces europaeiscabiei* strain NCPPB 4064	PRJNA255689	55.0	35.11	0.061
*Streptomyces* sp. EN16	PRJNA338712	54.9	34.79	0.061
*Streptomyces* sp. Root63	SAMN04155842	54.7	33.99	0.062
*Streptomyces* sp. Root1295	SAMN04155717	54.5	33.31	0.062
*Streptomyces* sp. W007	PRJNA74679	53.5	29.81	0.064
*Streptomyces griseus* subsp. griseus strain NRRL B-2682^T^	SAMN05558834	52.9	27.86	0.065
*Streptomyces* sp. OspMP-M43	SAMN04883176	52.6	27.01	0.066
*Streptomyces griseus* strain NRRL B-2165^T^	SAMN05558833	52.6	26.98	0.066
*Streptomyces* sp. MNU77	SAMN03200165	51.8	24.39	0.068
*Streptomyces griseus* subsp. griseus strain NRRL WC-3480	SAMN02645398	51.8	24.40	0.068


### Alkaline and Radio Tolerance Genes

The ability of bacteria such as *Streptomyces* sp. myrophorea, isolate McG1 to tolerate high levels of alkalinity is often attributed to alkaline shock genes, proton antiporters (like *nhaA*), and multidrug resistance factors (like *mdt*/*mdfA*) (Krulwich et al., 2011; Holdsworth and Law, 2013). We identified the presence of these genes in the annotated genomes of *Streptomyces* sp. myrophorea, isolate McG1 and *S. coelicolor* M145 listing 25 of these genes in *Streptomyces* sp. myrophorea, isolate McG1 and 9 genes in the genome of *S. coelicolor* M145 (**Table 4**). Only *Streptomyces* sp. myrophorea, isolate McG1 possessed alkaline shock genes, aspartate/ammonium antiporters, and multidrug transporters of the type *mdtH* (**Table 4**). Hence, the *Streptomyces* sp. was referred to as strain myrophorea, isolate McG1, from the Greek myro (fragrance; the isolate emits a strong fragrant odor) and phorea (porter/carrier; in recognition of the number of predicted antiporter genes).

**Table 4:** List of alkaline tolerance genes identified in *Streptomyces* sp. myrophorea, isolate McG1 and *S. coelicolor* M145.

*Streptomyces* sp. myrophorea, isolate McG1		*S. coelicolor* M145	
Gene		Gene	
504	Alkaline shock protein 23	HPPA_STRCO	K^+^ insensitive pyrophosphate
6561	Alkaline shock protein 23	Q9S2Y0_STRCO	Putative Na^+^/H^+^ antiporter
1049	Ammonium/H^+^ antiporter subunit *amhM*	Q9F3L8_STRCO	Putative Na^+^/H^+^ antiporter
2800	Aspartate/alanine antiporter	Q9KYW0_STRCO	Putative Na^+^/H^+^ antiporter
2801	Aspartate/alanine antiporter	Q9XAJ9_STRCO	Putative Na^+^/H^+^ antiporter
6218	Aspartate/alanine antiporter	NHAA1_STRCO	Na^+^/H^+^ antiporter *nhaA*
3284	Enhanced intracellular survival protein	NHAA2_STRCO	Na^+^/H^+^ antiporter *nhaA2*
159	K^+^/H^+^ antiporter *nhaP*	NHAA3_STRCO	Na^+^/H^+^ antiporter *nhaA3*
3788	K^+^/H^+^ antiporter *nhaP2*	Q93JF3_STRCO	Putative integral membrane ion exchanger
1050	K^+^/H^+^ antiporter *yhaU*		
2193	K^+^/H^+^ antiporter *yhaU*		
1325	K^+^ insensitive pyrophosphate-energized proton pump		
923	Multidrug resistance protein *mdtH*		
3240	Multidrug resistance protein *mdtH*		
3252	Multidrug resistance protein *mdtH*		
6781	Multidrug resistance protein *mdtH*		
1282	Na^+^/H^+^ antiporter *nhaA*		
4210	Na^+^/H^+^ antiporter subunit A		
4211	Na^+^/H^+^ antiporter subunit C		
4212	Na^+^/H^+^ antiporter subunit D		
4215	Na^+^/H^+^ antiporter subunit G		
660	Na^+^/(H^+^ or K^+^) antiporter *gerN*		
3425	Na^+^/(H^+^ or K^+^) antiporter *gerN*		
7210	Putative Na^+^/H^+^ exchanger		
1727	Sodium, potassium, lithium, and rubidium/H^+^ antiporter		


A similar identification of the number of DNA repair genes for *Streptomyces* sp. myrophorea, isolate McG1, and *S. coelicolor* M145 revealed little difference (**Supplementary Table S2**).

### Phylogeny

**FIGURE 4:**
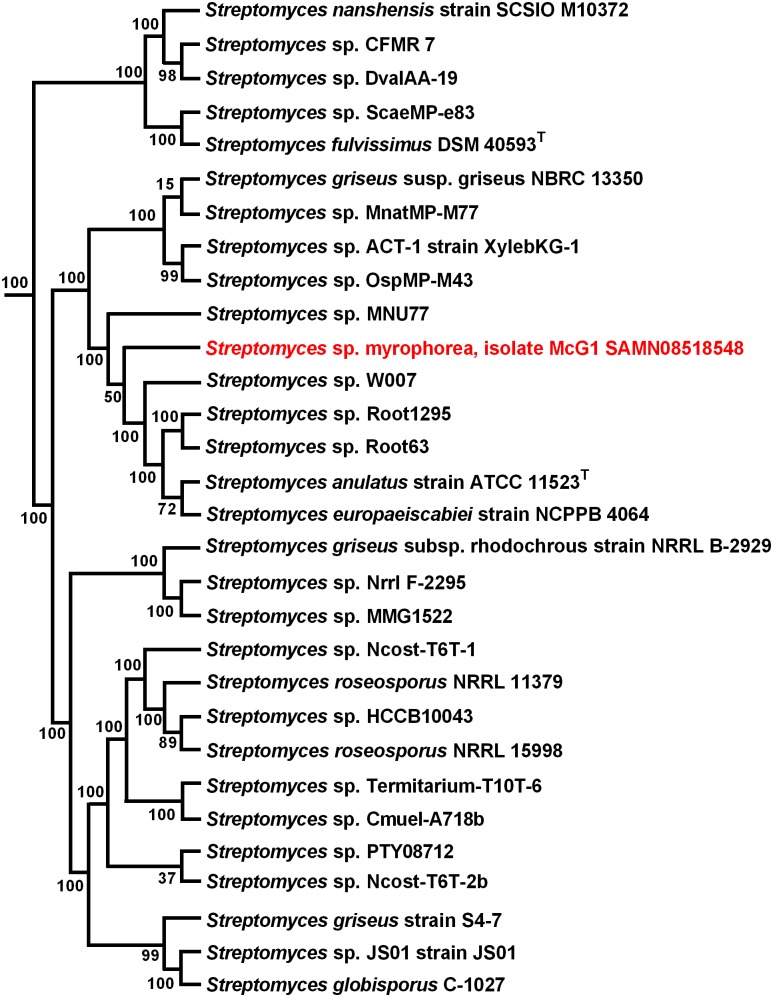
Maximum-likelihood phylogenetic tree of *Streptomyces* sp. myrophorea, isolate McG1. Tree shows the phylogenetic relationships between the closest GGDC matches; constructed with PhyloPhlAn. The phylogenetic tree was drawn in Fasttree from a concatenated alignment of 400 protein sequences in PhyloPhlAn. Numbers at nodes represent bootstrap values.

To infer the evolutionary history of *Streptomyces* sp. myrophorea, isolate McG1 a maximum-likelihood phylogeny was performed using a concatenation of 400 protein sequences (**Figure 4**).

### Identification of Secondary Metabolites

Potential antibiotic synthesis clusters from *Streptomyces* sp. myrophorea, isolate McG1 were predicted using anti-SMASH (Adamek et al., 2017). This revealed that *Streptomyces* sp. myrophorea, isolate McG1 possessed a total of 45 secondary metabolite biosynthesis gene clusters including multiple clusters with genes encoding the following secondary metabolite families: non-ribosomal peptide synthetase (NRPS) – 5 clusters; type I polyketide synthase (TI PKS) – 10 clusters; type III polyketide synthase (TIII PKS) – 2 clusters; terpenes – 5 clusters, lantipeptides – 3 clusters, and other biosynthesis genes clusters (BGCs) (**Table 5**).

**Table 5:** The distribution of biosynthesis gene clusters in the *Streptomyces* sp. myrophorea, isolate McG1.

Gene type	From	To	Biosynthetic gene cluster (BGC)
Aminoglycoside	211467	235061	Streptomycin BGC (19%)
Bacteriocin	60075	71379	–
Bacteriocin	58414	69211	Tetronasin BGC (3%)
Butyrolactone	86467	97372	Coelimycin BGC (8%)
Ectoine	67460	77858	Ectoine BGC (100%)
Ladderane-Arylpolyene-NRPS	171438	299095	Skyllamycin BGC (46%)
Lantipeptide	97251	120571	–
Lantipeptide	49038	71665	AmfS BGC (100%)
Melanin	98889	109311	Melanin BGC (100%)
Melanin	466637	477122	Melanin BGC (100%)
NRPS	121282	183145	Arixanthomycin BGC (5%)
NRPS	45983	133910	Coelichelin_BGC (90%)
NRPS	139743	190212	Griseoviridin_/_viridogrisein_BGC (5%)
NRPS	105944	148690	A54145_BGC (3%)
NRPS	1	38617	Friulimicin BGC (12%)
Other KS	103777	144736	Salinilactam BGC (8%)
Siderophore	55548	70283	–
Siderophore	8280	20058	Desferrioxamine B BGC (100%)
TI PKS	244352	272539	Kirromycin BGC (5%)
TI PKS	27526	57767	Nystatin-like pseudonocardia polyene BGC (26%)
TI PKS	1	9188	–
TI PKS	1	7809	Piericidin A1 BGC (50%)
TI PKS	1	42606	Kendomycin BGC (25%)
TI PKS	1	5761	Cremimycin BGC (17%)
TI PKS	1	7661	Nanchangmycin BGC (30%)
TI PKS	1	5579	–
TI PKS	1	22496	Piericidin A1 BGC (50%)
TI PKS	595702	666824	Lidamycin BGC (41%)
TI PKS-Linaridin	1	37834	Cypemycin BGC (77%)
TI PKS-NRPS	212061	264613	Daptomycin BGC (7%)
TI PKS-NRPS	8668	63347	Enduracidin BGC (8%)
TI PKS-NRPS	483977	533453	SGR_PTMs BGC (100%)
TI PKS-other KS	86013	144228	Borrelidin BGC (18%)
TIII PKS	201579	242697	Herboxidiene BGC (6%)
TIII PKS	509840	550892	Alkylresorcinol_ BGC (100%)
Terpene	51484	72797	2-Methylisoborneol BGC (100%)
Terpene	33012	55225	–
Terpene	309196	335766	Hopene BGC (69%)
Terpene	314534	335541	–
Terpene-NRPS	362776	437437	Isorenieratene BGC (100%)
Terpene-TI-PKS	1	44677	BE-7585A BGC (23%)
Thiopeptide	5614	38302	–
Trans-AT-PKS-other KS	40047	120375	Daptomycin BGC (6% )
Trans AT-PKS-TI PKS-Other KS, NRPS	356818	421396	–


*Biosynthesis gene clusters (%, indicates the proportion of genes showing similarity). TI PKS, Type I polyketide synthase; TIII PKS, Type III polyketide synthase; transAT-PKS, trans-amino transferase polyketide synthase; NRPS, non-ribosomal synthesized peptide; other KS, other ketide synthases.*

### *Streptomyces* sp. Myrophorea, Isolate McG1 Resistance to Antibiotics

**FIGURE 5:**
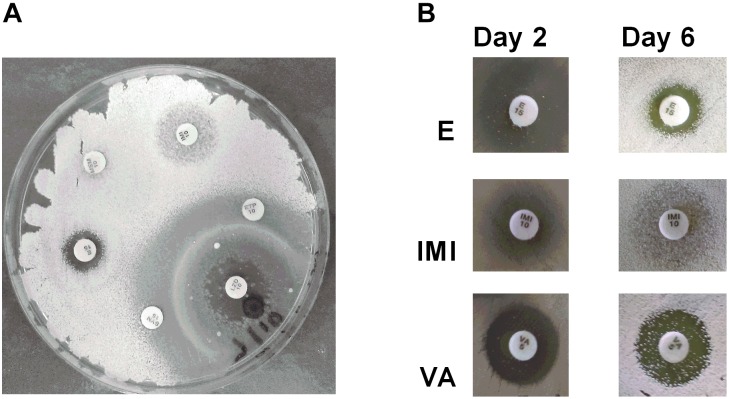
Antibiogram of *Streptomyces* sp. myrophorea, isolate McG1. **(A)** *Streptomyces* sp. myrophorea, isolate McG1 cultivated on ISP-2 agar (6 days) in the presence of an array of clinical antibiotics starting at the top the image and moving clockwise: imipenem (IMI 10), ertapenem (ETP 10), linezolid (LZD 10), quinpristin + dalropristin (SYN 15), erythromycin (E 15) and meropenem (MEM 10). **(B)** Delayed development of antibiotic resistance in *Streptomyces* sp. myrophorea, isolate McG1 only became apparent for some antibiotics after 6 days incubation in the case of erythromycin (E-15 μg), imipenem (IMI-10 μg), and vancomycin (VA-5 μg). The full antibiogram list is available in **Table 6**.

Antibiotic producing bacteria such as *Streptomyces* often require resistance genes to ameliorate the potentially toxic nature of their secondary metabolites. Such resistance elements can also be associated with antimicrobial biosynthesis gene clusters and can be used to predict potential antimicrobial synthesis (Nodwell, 2007). To better characterize these resistance elements, we cultivated *Streptomyces* sp. myrophorea, isolate McG1 in the presence of 36 different antibiotics (in triplicate). *Streptomyces* sp. myrophorea, isolate McG1 was resistant to 20 out of the 36 antibiotics tested after 2 days growth (**Table 6** and **Figure 5A**). Resistance to a further eight antibiotics was visually apparent after a further 4 days, most notably in vancomycin, produced by *Amycolatopsis orientalis*; imipenim, a β-lactam stablized version of thienamycin, produced by *Streptomyces cattleya* and erythromycin, a macrolide antibiotic produced by *Saccharopolyspora erythraea* (**Figure 5B**).

**Table 6:** Antibiogram of *Streptomyces* sp. myrophorea, isolate McG1.

Antibiotic	Amt (μg)	2 days	6 days
Amikacin	30	S	S
Ampicillin	20	R	R
Ampicillin + sulbactam	20	R	R
Augmentin	30	S	R
Apromycin	20	S	R
Carbenicillin	20	R	R
Cefepime	30	R	R
Ceftazidime	10	R	R
Ceftibuten	30	R	R
Cefoxitin	30	R	R
Ceftriaxone	30	R	R
Cefuroxime	30	R	R
Cefurin	5	R	R
Ciprofloxacin	5	I	R
Clavuranic acid	10	R	R
Ertapenem	10	R	R
Erythromycin	15	I	I
Gentamicin	10	S	S
Imipenem	10	S	R
Kanamycin	20	S	R
Linezolid	10	S	I
Meropenem	10	I	R
Mupiricin	200	R	R
Netilmicin	10	S	S
Nitrofurantoin	100	R	R
Novobiocin	5	S	R
Piperacillin + tazobactam	36	R	R
Quinpristin + dalropristin	5	R	R
Rifampicin	5	S	R
Streptomycin	20	R	R
Tigecycline	15	S	R
Tetracycline	20	R	R
Trimethoprim + sulfamethoxazole	25	R	R
Vancomycin	5	S	R


*Streptomyces sp. myrophorea, isolate McG1 was cultivated with clinical antibiotics. An inhibitory zone greater than 12 mm diameter was considered sensitive (S) to these antibiotics, a zone between 12 and 8 mm or a few colonies scattered in the inhibition zone was considered intermediate (I) and a zone of 8 mm diameter or less was considered resistant (R).*

### *In silico* Prediction of Antibiotic-Resistance Genes (ARGs) in *Streptomyces* sp. Myrophorea, Isolate McG1

Antibiotic resistance gene clusters for *Streptomyces* sp. myrophorea, isolate McG1 were predicted *in silico* using anti-SMASH. Many copies of multiple antibiotic resistance elements were also predicted through RAST including β-lactamases (classes A and C), metal-dependant hydrolase of β-lactams (metallo β-lactamase L1), β-lactamase (cephalosporinase), and other penicillin binding proteins (**Supplementary Table S3**). In addition many metal resistance elements were identified (through RAST) to mercury, copper, cobalt, zinc/cadmium, and arsenic which have been linked to increased antibacterial resistance (Chenia and Jacobs, 2017) (**Supplementary Table S3**). It is also possible that resistance may also be mediated through other mechanisms such as general multi-resistance clusters.

## Discussion

We have isolated a novel species of *Streptomyces* from an alkaline/radon environment that inhibits the growth of many multiresistant ESKAPE pathogens. There have been several reports in recent years of the presence of *Streptomyces* and other organisms in alkaline environments that can tolerate high pH levels (Tiago et al., 2004; Yucel and Yamac, 2010; Janto et al., 2011; Maciejewska et al., 2016). Our original hypothesis presumed that the Boho soil sample most likely contained *Streptomyces* which may produce antibiotics given the alkaline nature of the environment (Kontro et al., 2005). However, we were surprised to find that our isolate, *Streptomyces* sp. myrophorea, isolate McG1 inhibited the growth of many of the multiresistant ESKAPE pathogens. Some of these bacteria have been listed in a recent WHO document on priority pathogens urgently requiring the development of new antibiotics, such as carbapenem-resistant *A. baumannii* (at the top of this list) classified as a critical priority pathogen and vancomycin-resistant *Enterobacter faecium* and methicillin-resistant *S. aureus* classified as high priority pathogens (Tacconelli et al., 2018).

We have not ascertained the active component(s) responsible for inhibition of ESKAPE pathogens by *Streptomyces* sp. myrophorea, isolate McG1 as yet but this forms part of our ongoing research. Many *Streptomyces* species have the capacity to produce multiple antibiotics whose composition and identity can vary from species to species (Watve et al., 2001; Procopio et al., 2012). Given that our isolate encodes many antimicrobial gene clusters, it is entirely possible that some of these are responsible for the inhibition of ESKAPE pathogens. Given that our species is also novel and has the capacity to inhibit many multi-resistant pathogens also raises the possibility that some of its inhibitory components could be novel.

Another unexpected finding of the research was that although the pH of the Boho soil sample was around pH 8; our species of *Streptomyces* was able to grow at pH 10.5. This suggested not only tolerance of high alkaline conditions but the capacity to grow and divide in an extreme environments. More detailed tests will have to be made to ascertain the specifics of these optimal growth conditions.

The mechanism by which organisms such as *Streptomyces* can ameliorate high alkaline conditions is thought to be a product of a group of pH homeostasis genes, some of which have been identified in other species as alkaline shock genes, multidrug resistance factors (*mdt*/*mdfA*) and proton antiporters (*nhaA*) (Krulwich et al., 2011; Holdsworth and Law, 2013). Alkaline tolerance is also linked to the production of antibiotics through the presence of multidrug resistance factors which are often associated with antimicrobial biosynthesis gene clusters (Nodwell, 2007).

In support of our results, genome sequencing of *Streptomyces* sp. myrophorea, isolate McG1 revealed that it contained more annotated alkaline tolerance genes than *S. coelicolor* M145 (at 25/9). Furthermore alkaline shock genes, ammonium and aspartate antiporters and multidrug resistance antiporters of the type *mdt*H were only identified in the *Streptomyces* sp. myrophorea, isolate McG1 genome and not in *S. coelicolor* M145 genome. Indeed, it was this abundance of porters and the fragrant smell of the *Streptomyces* that prompted us to refer to the strain as myrophorea, isolate McG1, myro (Greek for fragrance) and phorea (Greek for porter).

The sequencing of the *Streptomyces* sp. myrophorea, isolate McG1 genome also enabled the prediction of potential antibiotics through anti-Smash and RAST (Aziz et al., 2008; Medema et al., 2011). However, given the cryptic nature of many *Streptomyces* spp. metabolites, it cannot be assumed these antibiotics are produced until their products are identified. We were also able to predict many antibiotic resistance clusters through RAST which are often associated with antimicrobial biosynthesis gene clusters (Nodwell, 2007). Antimicrobial resistance predictions were followed by *in vitro* experiments on 36 clinical antibiotics. This data showed that *Streptomyces* sp. myrophorea, isolate McG1 was resistant to 28 out of 36 antibiotics. Specifically it was resistant to nearly all the β-lactams, with the exception of augmentin to which resistance developed after 6 days. The same pattern of delayed resistance was seen for glycopeptides, fluoroquinones (such as ciprofloxacin), and tetracyclines (such as tigecycline).

It is interesting that the ancient healers of Boho made a connection between alkaline soils (containing *Streptomyces* sp.) and (skin) infections. It has only recently been discovered that the pH of the specific infection sites can rise from normal skin pH about pH 5.5 to 8.5 and that bacterial biofilms, which can colonize wounds, can also reach similar pH levels (Schneider et al., 2007; Hostacka et al., 2010; Percival et al., 2014). Perhaps the indigenous people, who were undoubtedly in close proximity to the soil, noted (after many years) its curative properties under specific conditions. However, it is difficult to know the exact genesis of this cure because the previous occupants of the Boho site, the Druids, left no surviving records of healing and their Neolithic counterparts left only undeciphered carvings on some nearby stones. Anthropologically, the Boho folk tradition is similar to that of Kisameet Bay clay in Canada, another indigenous soil cure which was found to have inhibitory activity against a range of ESKAPE pathogens (Behroozian et al., 2016).

## Conclusion

We have isolated and genome sequenced a novel alkaline and radio-tolerant species of *Streptomyces* from an ethnopharmacological soil cure; *Streptomyces* sp. myrophorea, isolate McG1. This *Streptomyces* sp. inhibits many multi-resistant ESKAPE pathogens including carbapenem-resistant *A. baumannii* (a critical priority species from the WHO priority list of antibiotic-resistant bacteria), vancomycin-resistant *E. faecium*, and methicillin-resistant *S. aureus* (listed as high priority by the WHO). Although not a complete elucidation of the antibacterial components of the Boho soil; we think that inhibition of such pathogens by *Streptomyces* sp. myrophorea, isolate McG1 may explain some of its reputed curative properties. It is hoped to further characterize some of these inhibitory components from *Streptomyces* sp. myrophorea, isolate McG1 and investigate the properties of other species contained in alkaline soil. We hope this will advance progress in stemming the tide of multi-resistant bacteria.

## Data Availability

All datasets (GENERATED/ANALYZED) for this study are included in the manuscript and the **Supplementary Files**.

## Author Contributions

The idea for the isolation of extreme *Streptomyces* was an offshoot from the work of PD and LuT. Microbiology, strain growth, and testing were by GQ, LuT, DV, and IB. Genomic isolation and sequencing by AA and MH. Bioinformatics by PF, MH, PD, LiT, and AA. Imaging by SG and LF. Manuscript ideas and editing of relevant sections by all authors.

## Conflict of Interest Statement

The authors declare that the research was conducted in the absence of any commercial or financial relationships that could be construed as a potential conflict of interest.

## Footnotes

1 http://www.eucast.org

2 http://rast.nmpdr.org

3 https://www.ncbi.nlm.nih.gov/genome/genomes/13511

## References

[B1] Adamek M. Spohn M. Stegmann E. Ziemert N. (2017). Mining bacterial genomes for secondary metabolite gene Clusters. *Methods Mol. Biol.* 1520 23–47. 27873244 10.1007/978-1-4939-6634-9_2

[B2] Auch A. F. von Jan M. Klenk H.-P. Göker M. (2010). Digital DNA-DNA hybridization for microbial species delineation by means of genome-to-genome sequence comparison. *Stand. Genomic Sci.* 2 117–134. 10.4056/sigs.531120 21304684 PMC3035253

[B3] Aziz R. K. Bartels D. Best A. A. DeJongh M. Disz T. Edwards R. A. (2008). The RAST server: rapid annotations using subsystems technology. *BMC Genomics* 9:75. 10.1186/1471-2164-9-75 18261238 PMC2265698

[B4] Bankevich A. Nurk S. Antipov D. Gurevich A. A. Dvorkin M. Kulikov A. S. (2012). SPAdes: a new genome assembly algorithm and its applications to single-cell sequencing. *J. Comput. Biol.* 19 455–477. 10.1089/cmb.2012.0021 22506599 PMC3342519

[B5] Bauer A. W. Kirby W. M. Sherris J. C. Turck M. (1966). Antibiotic susceptibility testing by a standardized single disk method. *Am. J. Clin. Pathol.* 45 493–496. 10.1093/ajcp/45.4_ts.493 5325707

[B6] Behroozian S. Svensson S. L. Davies J. (2016). Kisameet clay exhibits potent antibacterial activity against the eskape pathogens. *mBio* 7 e1842–e1815. 10.1128/mBio.01842-15 26814180 PMC4742703

[B7] Bolger A. M. Lohse M. Usadel B. (2014). Trimmomatic: a flexible trimmer for illumina sequence data. *Bioinformatics* 30 2114–2120. 10.1093/bioinformatics/btu170 24695404 PMC4103590

[B8] Brunton C. H. C. Mason T. R. (1979). Palaeoenvironments and correlation of the carboniferous rocks in West Fermanagh. Northern Ireland. *Bull. Br. Mus. Nat. Hist. Geol.* 32 91–108.

[B9] Chenia H. Y. Jacobs A. (2017). Antimicrobial resistance, heavy metal resistance and integron content in bacteria isolated from a South African tilapia aquaculture system. *Dis. Aquat. Organ.* 126 199–209. 10.3354/dao03173 29160218

[B10] Daly M. J. (2012). Death by protein damage in irradiated cells. *DNA Repair.* 11 12–21. 10.1016/j.dnarep.2011.10.024 22112864

[B11] Daraktchieva Z. Appleton J. D. Rees D. M. Adlam K. A. M. Myers A. H. Hodgson S. A. (2015). *Radon in Northern Ireland: Indicative Atlas*. Available at: https://www.gov.uk/government/publications/radon-indicative-atlas-for-northern-ireland

[B12] Donnelly C. MacDonald P. Murphy E. Beer N. (2003). Excavations at boho high cross, toneel north, county fermanagh. *Ulst. J. Archaeol.* 62 121–142.

[B13] Foley R. (2015). Indigenous narratives of health: (Re)placing folk-medicine within irish health histories. *J. Med. Humanit.* 36 5–18. 10.1007/s10912-014-9322-4 25483616 PMC4352604

[B14] Founou R. C. Founou L. L. Essack S. Y. (2017). Clinical and economic impact of antibiotic resistance in developing countries: a systematic review and meta-analysis. *PLoS One* 12:e0189621. 10.1371/journal.pone.0189621 29267306 PMC5739407

[B15] Gallachair P. Ó. (1975). Clogherici: a dictionary of the catholic clergy of the diocese of clogher (1535-1835) (continued). *Clogher Rec.* 8 271–280. 10.2307/27695713

[B16] Gurevich A. Saveliev V. Vyahhi N. Tesler G. (2013). QUAST: quality assessment tool for genome assemblies. *Bioinformatics* 29 1072–1075. 10.1093/bioinformatics/btt086 23422339 PMC3624806

[B17] Halpin A. Newman C. (2009). *Ireland: An Oxford Archaeological Guide to Sites from Earliest Times to AD 1600*. Oxford: Oxford Univ. Press.

[B18] Holdsworth S. R. Law C. J. (2013). Multidrug resistance protein MdtM adds to the repertoire of antiporters involved in alkaline pH homeostasis in *Escherichia coli*. *BMC Microbiol.* 13:113. 10.1186/1471-2180-13-113 23701827 PMC3668916

[B19] Hostacka A. Ciznar I. Stefkovicova M. (2010). Temperature and pH affect the production of bacterial biofilm. *Folia Microbiol.* 55 75–78. 10.1007/s12223-010-0012-y 20336508

[B20] Janto B. Ahmed A. Ito M. Liu J. Hicks D. B. Pagni S. (2011). The genome of alkaliphilic *Bacillus pseudofirmus* OF4 reveals adaptations that support the ability to grow in an external ph range from 7.5 to 11.4. *Environ. Microbiol.* 13 3289–3309. 10.1111/j.1462-2920.2011.02591.x 21951522 PMC3228905

[B21] Jelic M. Butic I. Plecko V. Cipris I. Jajic I. Bejuk D. (2016). KPC-Producing *Klebsiella pneumoniae* isolates in croatia: a nationwide survey. *Microb. Drug Resist.* 22 662–667. 10.1089/mdr.2015.0150 26709956

[B22] Jelic M. Skrlin J. Bejuk D. Koscak I. Butic I. Guzvinec M. (2017). Characterization of isolates associated with emergence of OXA-48-producing *Klebsiella pneumoniae* in croatia. *Microb. Drug Resist.* 24 973–979. 10.1089/mdr.2017.0168 29267137

[B23] Jones S. E. Ho L. Rees C. A. Hill J. E. Nodwell J. R. Elliot M. A. (2017). Streptomyces exploration is triggered by fungal interactions and volatile signals. *eLife* 6:e21738. 10.7554/eLife.21738 28044982 PMC5207766

[B24] Kontro M. Lignell U. Hirvonen M.-R. Nevalainen A. (2005). pH effects on 10 *Streptomyces* spp. growth and sporulation depend on nutrients. *Lett. Appl. Microbiol.* 41 32–38. 10.1111/j.1472-765X.2005.01727.x 15960749

[B25] Krulwich T. A. Sachs G. Padan E. (2011). Molecular aspects of bacterial pH sensing and homeostasis. *Nat. Rev. Microbiol.* 9 330–343. 10.1038/nrmicro2549 21464825 PMC3247762

[B26] Kung S. H. Lund S. Murarka A. McPhee D. Paddon C. J. (2018). Approaches and recent developments for the commercial production of semi-synthetic artemisinin. *Front. Plant Sci.* 9:87. 10.3389/fpls.2018.00087 29445390 PMC5797932

[B27] Lehrer R. I. Rosenman M. Harwig S. S. Jackson R. Eisenhauer P. (1991). Ultrasensitive assays for endogenous antimicrobial polypeptides. *J. Immunol. Methods* 137 167–173. 10.1016/0022-1759(91)90021-7 1901580

[B28] Lewis K. (2013). Platforms for antibiotic discovery. *Nat. Rev. Drug Discov.* 12 371–387. 10.1038/nrd3975 23629505

[B29] Li B. Webster T. J. (2018). Bacteria antibiotic resistance: new challenges and opportunities for implant-associated orthopaedic infections. *J. Orthop. Res.* 36 22–32. 10.1002/jor.23656 28722231 PMC5775060

[B30] Maciejewska M. Adam D. Martinet L. Naome A. Calusinska M. Delfosse P. (2016). A phenotypic and genotypic analysis of the antimicrobial potential of cultivable *Streptomyces* isolated from cave moonmilk deposits. *Front. Microbiol.* 7:1455. 10.3389/fmicb.2016.01455 27708627 PMC5030222

[B31] Mao J. Tang Q. Zhang Z. Wang W. Wei D. Huang Y. (2007). *Streptomyces radiopugnans* sp. nov., a radiation-resistant actinomycete isolated from radiation-polluted soil in China. *Int. J. Syst. Evol. Microbiol.* 57 2578–2582. 10.1099/ijs.0.65027-0 17978221

[B32] Medema M. H. Blin K. Cimermancic P. de Jager V. Zakrzewski P. Fischbach M. A. (2011). antiSMASH: rapid identification, annotation and analysis of secondary metabolite biosynthesis gene clusters in bacterial and fungal genome sequences. *Nucleic Acids Res.* 39 W339–W346. 10.1093/nar/gkr466 21672958 PMC3125804

[B33] Miller M. A. Pfeiffer W. Schwartz T. (2010). “Creating the CIPRES science gateway for inference of large phylogenetic trees,” in *2010 Gateway Computing Environments Workshop (GCE)*, New Orleans, LA, 1–8. 10.1109/GCE.2010.5676129

[B34] Mohammadipanah F. Wink J. (2015). Actinobacteria from arid and desert habitats: diversity and biological activity. *Front. Microbiol.* 6:1541 10.3389/fmicb.2015.01541 PMC4729944 26858692

[B35] Nguyen T. M. Kim J. (2015). Antifungal and antibacterial activities of *Streptomyces polymachus* sp. nov. Isolated from soil. *Int. J. Syst. Evol. Microbiol.* 65 2385–2390. 10.1099/ijs.0.000268 25899502

[B36] Nkanga E. J. Hagedorn C. (1978). Detection of antibiotic-producing streptomyces inhabiting forest soils. *Antimicrob. Agents Chemother.* 14 51–59. 10.1128/AAC.14.1.51 686709 PMC352404

[B37] Nodwell J. R. (2007). Novel links between antibiotic resistance and antibiotic production. *J. Bacteriol.* 189 3683–3685. 10.1128/JB.00356-07 17384190 PMC1913332

[B38] Noomnual S. Thasana N. Sungkeeree P. Mongkolsuk S. Loprasert S. (2016). Streptanoate, a new anticancer butanoate from *Streptomyces* sp. DC3. *J. Antibiot.* 69 124–127. 10.1038/ja.2015.95 26374563

[B39] Percival S. L. McCarty S. Hunt J. A. Woods E. J. (2014). The effects of pH on wound healing, biofilms, and antimicrobial efficacy. *Wound Repair Regen.* 22 174–186. 10.1111/wrr.12125 24611980

[B40] Procopio R. E. Silva I. R. Martins M. K. Azevedo J. L. Araújo J. M. (2012). Antibiotics produced by *Streptomyces*. *Braz. J. Infect. Dis.* 16 466–471. 10.1016/j.bjid.2012.08.014 22975171

[B41] Santajit S. Indrawattana N. (2016). Mechanisms of antimicrobial resistance in ESKAPE pathogens. *Biomed Res. Int.* 2016:2475067. 10.1155/2016/2475067 27274985 PMC4871955

[B42] Sato M. Beppu T. Arima K. (1983). Studies on antibiotics produced at high alkaline pH. *Agric. Biol. Chem.* 47 2019–2027. 10.1080/00021369.1983.10865910

[B43] Schatz A. Bugie E. Waksman S. (1944). Streptomycin: a substance exhibiting antibiotic activity against gram-positive and gram-negative bacteria. *Proc. Soc. Exp. Biol. Med.* 55 66–69. 10.3181/00379727-55-14461 16056018

[B44] Schneider L. A. Korber A. Grabbe S. Dissemond J. (2007). Influence of pH on wound-healing: a new perspective for wound-therapy? *Arch. Dermatol. Res.* 298 413–420. 10.1007/s00403-006-0713-x 17091276

[B45] Seemann T. (2014). Prokka: rapid prokaryotic genome annotation. *Bioinformatics* 30 2068–2069. 10.1093/bioinformatics/btu153 24642063

[B46] Segata N. Bornigen D. Morgan X. C. Huttenhower C. (2013). PhyloPhlAn is a new method for improved phylogenetic and taxonomic placement of microbes. *Nat. Commun.* 4:2304. 10.1038/ncomms3304 23942190 PMC3760377

[B47] Tacconelli E. Carrara E. Savoldi A. Harbarth S. Mendelson M. Monnet D. L. (2018). Discovery, research, and development of new antibiotics: the WHO priority list of antibiotic-resistant bacteria and tuberculosis. *Lancet Infect. Dis.* 18 318–327. 10.1016/S1473-3099(17)30753-3 29276051

[B48] Tiago I. Chung A. P. Veríssimo A. (2004). Bacterial diversity in a nonsaline alkaline environment: heterotrophic aerobic populations. *Appl. Environ. Microbiol.* 70 7378–7387. 10.1128/AEM.70.12.7378-7387.2004 15574939 PMC535156

[B49] Watve M. G. Tickoo R. Jog M. M. Bhole B. D. (2001). How many antibiotics are produced by the genus *Streptomyces*? *Arch. Microbiol.* 176 386–390. 10.1007/s002030100345 11702082

[B50] Yokomizo K. Miyamoto Y. Nagao K. Kumagae E. Habib E. S. Suzuki K. (1998). Fattiviracin A1, a novel antiviral agent produced by *Streptomyces microflavus* strain No. 2445. II. Biological properties. *J. Antibiot.* 51 1035–1039. 10.7164/antibiotics.51.1035 9918397

[B51] Yucel S. Yamac M. (2010). Selection of *Streptomyces* isolates from turkish karstic caves against antibiotic resistant microorganisms. *Pak. J. Pharm. Sci.* 23 1–6. 20067859

