# Supplementary material for: A Novel Alkaliphilic Streptomyces Inhibits ESKAPE Pathogens
Source: Front Microbiol. 2018 Oct 16;9:2458. doi: 10.3389/fmicb.2018.02458 (PMC6232825; doi:10.3389/fmicb.2018.02458)
Supplement: Supplementary file 1 [file Table_1.DOCX]

Supplementary Material

A Novel Alkaliphilic Streptomyces inhibits ESKAPE Pathogens.

**Luciana Terra^1^, Paul J Dyson^1^, Matthew D Hitchings^1^, Liam Thomas^1^, Alyaa Abdelhameed^1^, Ibrahim M Banat^2^, Salvatore A Gazze^1^, Dušica Vujaklija^3^, Paul D Facey^1^, Lewis W Francis^1^, Gerry A. Quinn^3^****

**Correspondence:** Dr. Gerry A. Quinn gquinn@irb.hr

**TABLE S1. Antimicrobial sensitivity test for ESKAPE, ATCC bacterial strains (Kirby-Bauer method**). Antibiotic (discs) used in sensitivity testing (Oxoid). Amount of antibiotic in the discs (in µg) given after antibiotic abbreviation. Sensitive to the antibiotic (S), resistant to antibiotic (R). Amikacin (AK 30), amoxicillin-clavulanate 2:1 (AMC 30), ampicillin (Amp 10), amoxicillin (AMX 10) , ceftazidime (CAZ 10), cefuroxime (CFX 30), ciprofloxacin (CIP 5), clindamycin (CLI 10), Cefepime (CPM 30), Colistin (CST 10), Cefotaxime (30 CTX), Cefuroxime (CXM 30), ertapenem (ETP 10), Cefoxitin (FOX 30), Gentamicin (GEN 10), imipenem (IMI 10), linezolid (LZD 10), meropenem (MEM 10), Oxacillin (OXA 5), Piperacillin + tazobactam (PTZ) 36), Rifampicin (RP 5), Ampicillin/Sulbactam (SAM 20), Trimethoprim + sulfamethoxazole (SXT/TS 25), teicoplanin (TEC 30), Vancomycin (VAN 5). WHO critical-priority bacteria (*) WHO high priority bacteria (**).

| **Bacterial strain** | **Antimicrobial sensitivity test** | | | | | | | | | | |  |
| --- | --- | --- | --- | --- | --- | --- | --- | --- | --- | --- | --- | --- |
| *Enterococcus faecium (a)* | AMP R | VAN S | TEC S | LZD S | GEN S |  |  |  |  |  |  | |
| *E. faecium (b)* | AMP S | VAN S |  | LZD S | GEN R |  |  |  |  |  |  | |
| *E. faecium (c) VRE*** | AMP R | VAN R | TEC R | LZD S | GEN R |  |  |  |  |  |  | |
| *Staphylococcus aureus (a)* | FOX S | RP S | TEC S | LZD S | OXA S | VAN S | CTX S | CIP S |  |  |  | |
| *S. aureus(b) (MRSA)*** | FOX R | CIP R | OXA R | RP S | LZD S | CTX S |  |  |  |  |  | |
| *S. aureus (e) (MRSA)*** | FOX R | CIP S | RP S | LZD S | CLI S | CTX S | VAN S. | OXA R |  |  |  | |
| *Klebsiella pneumonia* (a) | GEN S | CIP S | AK S | CTX S | CAZ S | IMI S | MEM S. |  |  |  |  | |
| *K. pneumonia* (b) | GEN S | CIP S | AK S | CTX S | CAZ S | IMI S | MEM S. |  |  |  |  | |
| *K. pneumonia*(d) | AMP R | AK S | CIP S | CTX S | CAZ S | CTX S | IMI S  MEM S | GEN S | AMC S | CPM S |  | |
| *Acinetobacter baumanii* (a)* | SAM S | MEM R | CIP R | GEN R | AK R | CTX R | CST S | IMI R |  |  |  | |
| *A. baumanii* (d)* | SAM S | MEM R | CIP R | GEN R | AK R | CTX R | CST S. | IMI R |  |  |  | |
| *A. baumanii* (e)* | SAM S | MEM R | CIP R | GEN R | AK R | CTX R | CST S | IMI R |  |  |  | |
| *Pseudomonas aeruginosa* (a) | PTZ S | GEN S | AK S | CIP S | CAZ S | IMI S | MEM S |  |  |  |  | |
| *P. aeruginosa* (b) | PTZ S | GEN S | AK S | CIP S | CAZ S | IMI S. | MEM S |  |  |  |  | |
| *Enterobacter cloacae* (a) | AMX R | AMC R | PTZ R | CFX R | CXM R | CTX R | CAZ R | CPM S | ETP R  IMI S  MEM S | GEN R  AK S | SXT R  CIP R | |
