# Supplementary material for: A Novel Alkaliphilic Streptomyces Inhibits ESKAPE Pathogens
Source: Front Microbiol. 2018 Oct 16;9:2458. doi: 10.3389/fmicb.2018.02458 (PMC6232825; doi:10.3389/fmicb.2018.02458)
Supplement: Supplementary file 2 [file Table_2.DOCX]

Supplementary Material

A Novel Alkaliphilic Streptomyces inhibits ESKAPE Pathogens.

**Luciana Terra^1^, Paul J Dyson^1^, Matthew D Hitchings^1^, Liam Thomas^1^, Alyaa Abdelhameed^1^, Ibrahim M Banat^2^, Salvatore A Gazze^1^, Dušica Vujaklija^3^, Paul D Facey^1^, Lewis W Francis^1^, Gerry A. Quinn^3^****

**Correspondence:** Dr. Gerry A. Quinn gquinn@irb.hr

**TABLE S2. The annotation of DNA repair proteins in the genomes of *Streptomyces* sp. myrophorea, isolate McG1** **and *S. coelicolor* M145.**

| ***Streptomyces* sp. strain myrophorea, isolate McG1** | | ***S. coelicolor* M145** | |
| --- | --- | --- | --- |
| **Gene** | **DNA repair protein** | **Gene** | **DNA repair protein** |
| **3695** | Protein ***recA*** | **RECA_STRCO** | Protein ***recA*** |
| **784** | ***recBCD*** enzyme subunit ***recD*** |  |  |
| **4533** | ***recBCD*** enzyme subunit ***recD*** |  |  |
| **4003** | ATP-dependent ***recD*-**like DNA helicase | Q9RDI9_STRCO | ATP-dependent recD-like DNA helicase |
| **1163** | DNA replication and repair protein ***recF*** | **RECF_STRCO** | DNA replication and repair protein ***recF*** |
| **1298** | DNA replication and repair protein ***recF*** |  |  |
| **4662** | DNA repair protein ***RecN*** | **RECN_STRCO** | DNA repair protein ***RecN*** |
| **7588** | ATP-dependent DNA helicase ***recG*** | **Q9ZBR3_STRCO** | ATP-dependent DNA helicase ***recG*** |
| **3736** | ATP-dependent DNA helicase ***recQ*** |  |  |
| **5186** | ATP-dependent DNA helicase ***recQ*** |  |  |
| **2084** | DNA repair protein ***recO*** |  |  |
| **1186** | Recombination protein ***recR*** | **RECR_STRCO** | Recombination protein ***recR*** |
| **3696** | Regulatory protein ***recX*** | **RECX_STRCO** | Regulatory protein ***recX*** |
