# Supplementary material for: A Novel Alkaliphilic Streptomyces Inhibits ESKAPE Pathogens
Source: Front Microbiol. 2018 Oct 16;9:2458. doi: 10.3389/fmicb.2018.02458 (PMC6232825; doi:10.3389/fmicb.2018.02458)
Supplement: Supplementary file 3 [file Table_3.DOCX]

Supplementary Material

A Novel Alkaliphilic Streptomyces inhibits ESKAPE Pathogens.

**Luciana Terra^1^, Paul J Dyson^1^, Matthew D Hitchings^1^, Liam Thomas^1^, Alyaa Abdelhameed^1^, Ibrahim M Banat^2^, Salvatore A Gazze^1^, Dušica Vujaklija^3^, Paul D Facey^1^, Lewis W Francis^1^, Gerry A. Quinn^3^****

**Correspondence:** Dr. Gerry A. Quinn [gquinn@irb.hr](mailto:gquinn@irb.hr)

**TABLE S3. The distribution of resistance genes in *Streptomyces* sp. myrophorea, isolate McG1** **genome**

| Subsystem | Role |
| --- | --- |
| Copper homeostasis | Multicopper oxidase |
| Copper homeostasis | Copper-translocating P-type ATPase (EC 3.6.3.4) |
| Copper homeostasis | Copper chaperone |
| Copper homeostasis | Copper resistance protein D |
| Cobalt-zinc-cadmium resistance | DNA-binding heavy metal response regulator |
| Cobalt-zinc-cadmium resistance | Cobalt-zinc-cadmium resistance protein CzcD |
| Cobalt-zinc-cadmium resistance | Probable Co/Zn/Cd efflux system membrane fusion protein |
| Cobalt-zinc-cadmium resistance | Transcriptional regulator, MerR family |
| **Resistance to Vancomycin** | VAN B-type resistance protein VanW |
| **Resistance to Vancomycin** | VAN response regulator VanR |
| Mercuric reductase | FAD-dependent NAD(P)-disulphide oxidoreductase |
| Arsenic resistance | Arsenical-resistance protein ACR3 |
| Arsenic resistance | Arsenical resistance operon repressor |
| Arsenic resistance | Arsenical pump-driving ATPase (EC 3.6.3.16) |
| Arsenic resistance | Arsenic efflux pump protein |
| Arsenic resistance | Arsenate reductase (EC 1.20.4.1) |
| Copper homeostasis: copper tolerance | Cytoplasmic copper homeostasis protein CutC |
| Copper homeostasis: copper tolerance | Magnesium and cobalt efflux protein CorC |
| **Resistance to fluoroquinolones** | DNA gyrase subunit B (EC 5.99.1.3) |
| **Resistance to fluoroquinolones** | DNA gyrase subunit A (EC 5.99.1.3) |
| **Resistance to fluoroquinolones** | Topoisomerase IV subunit B (EC 5.99.1.-) |
| **Resistance to fluoroquinolones** | Topoisomerase IV subunit A (EC 5.99.1.-) |
| **β -lactamase** | β-lactamase class A |
| **β -lactamase** | β-lactamase class C and other penicillin binding proteins |
| **β -lactamase** | β-lactamase (EC 3.5.2.6) |
| **β -lactamase** | Metal-dependent hydrolases of the β-lactamase superfamily III |
|  |  |
